# Association between breastfeeding duration and diabetes mellitus in menopausal women: a machine-learning analysis using population-based retrospective study

**DOI:** 10.1186/s13006-024-00642-z

**Published:** 2024-05-14

**Authors:** Eun-Saem Choi, Jue Seong Lee, Hwasun Lee, Kwang-Sig Lee, Ki Hoon Ahn

**Affiliations:** 1 Department of Obstetrics & Gynecology, Korea University College of Medicine, Korea University Anam Hospital, 73 Goryeodae-ro, Seongbuk-gu, Seoul, 02841 Korea; 2 Department of Pediatrics, Korea University College of Medicine, Korea University Anam Hospital, Seoul, Korea; 3 Department of Biostatistics, Korea University College of Medicine, Seoul, Korea; 4 AI Center, Korea University College of Medicine, Korea University Anam Hospitald, 73 Goryeodae-ro, Seongbuk-gu, Seoul, 02841 Korea

**Keywords:** Diabetes, Breastfeeding, Prevention, Machine learning

## Abstract

**Background:**

Breastfeeding resets insulin resistance caused by pregnancy however, studies on the association between breastfeeding and diabetes mellitus (DM) have reported inconsistent results. Therefore, we aimed to investigate the risk of DM according to breastfeeding duration in large-scale population-based retrospective study. In addition, machine-learning prediction models for DM and hemoglobin A1c (HbA1c) were developed to further evaluate this association.

**Methods:**

We used the Korean National Health and Nutrition Examination Surveys database, a nationwide and population-based health survey from 2010 to 2020. We included 15,946 postmenopausal women with a history of delivery, whom we divided into three groups according to the average breastfeeding duration: (1) no breastfeeding, (2) < 12 months breastfeeding, and (3) ≥ 12 months breastfeeding. Prediction models for DM and HbA1c were developed using an artificial neural network, decision tree, logistic regression, Naïve Bayes, random forest, and support vector machine.

**Results:**

In total, 2248 (14.1%) women had DM and 14,402 (90.3%) had a history of breastfeeding. The prevalence of DM was the lowest in the < 12 breastfeeding group (no breastfeeding vs. < 12 months breastfeeding vs. ≥ 12 months breastfeeding; 161 [10.4%] vs. 362 [9.0%] vs. 1,725 [16.7%], *p *< 0.001). HbA1c levels were also the lowest in the < 12 breastfeeding group (HbA1c: no breastfeeding vs. < 12 months breastfeeding vs. ≥ 12 months breastfeeding; 5.9% vs. 5.9% vs. 6.1%, respectively, *p *< 0.001). After adjustment for covariates, the risk of DM was significantly increased in both, the no breastfeeding (adjusted odds ratio [aOR] 1.29; 95% CI 1.29, 1.62]) and ≥ 12 months of breastfeeding groups (aOR 1.18; 95% CI 1.01, 1.37) compared to that in the < 12 months breastfeeding group. The accuracy and the area under the receiver-operating-characteristic curve of the DM prediction model were 0.93 and 0.95, respectively. The average breastfeeding duration was ranked among the top 15 determinants of DM, which supported the strong association between breastfeeding duration and DM. This association was also observed in a prediction model for HbA1c.

**Conclusions:**

Women who did not breasted had a higher risk of developing DM than those who breastfed for up to 12 months.

**Supplementary Information:**

The online version contains supplementary material available at 10.1186/s13006-024-00642-z.

## Background

Breastfeeding is known to not only decrease the risk of neonatal/infant morbidity and mortality, but also have beneficial effects on maternal health [1–7]. Breastfeeding is reported to help the resetting process of metabolic changes caused by pregnancy which includes insulin resistance [8]. However, the results of previous studies on breastfeeding and maternal risk of diabetes mellitus (DM) are not consistent. Some studies which have demonstrated a possible risk reduction of maternal DM, hypertension, and metabolic diseases [9–13], while others have found no association between breastfeeding and DM [14–18]. There could be various reasons for such inconsistent results, but factors such as the duration of breastfeeding or parity could be the potential causes. In previous studies, the duration of breastfeeding varied, and some studies analyzed the association between breastfeeding and DM without considering the duration of breastfeeding [14, 16–19].

In this study, we aimed to investigate the risk of DM according to breastfeeding duration in a large-scale population-based retrospective design in South Korea. Furthermore, we used machine learning to optimize the analysis of large-scale data containing a large number of confounding variables for diabetes, including medical history; obstetric characteristics; and demographic, socioeconomic, and lifestyle characteristics. We developed a prediction model for DM and hemoglobin A1c (HbA1c) using machine learning and evaluated the association between breastfeeding duration and DM.

## Methods

### Participants

**Fig. 1:**
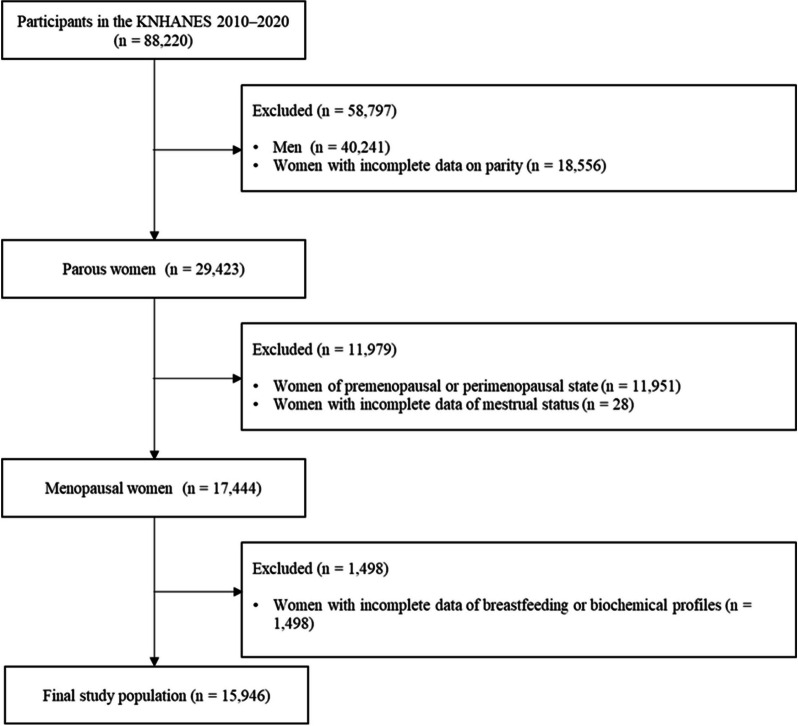
Flow diagram of the study population

This retrospective cross-sectional study used data from the Korean National Health and Nutrition Examination Surveys (KNHANES) database. The KNHANES, a nationwide and population-based health survey, was performed by the Korean Ministry of Health and Welfare. The KNHANES included a health examination, health interview, and nutrition survey. The KNHANES measured anthropometric measures and biochemical profiles from the participants’ blood and urine samples. The information on socioeconomic status and health behaviors were also collected through the interview and surveys and the interviews and surveys were conducted by well-trained researchers. The KNHANES has been described in previous studies [20]. The current study used data from the fifth (2010–2012), sixth (2013–2015), seventh (2016–2018), or eighth (2019–2020) KNHANES. Among the KNHANES participants, postmenopausal women with a history of delivery were selected as the study population. Women with incomplete data on gravidity, breastfeeding, or biochemical profiles were excluded, and 15,946 postmenopausal/parous women were included in the final analysis (Fig. 1).

### Variables

This study considered various covariates, including sociodemographic characteristics, lifestyle characteristics, medical and obstetric history (including breasting duration), and biochemical profiles (Table 1). Data regarding sociodemographic characteristics, health-related variables, and medical and obstetric histories (including breasting duration) were obtained from the questionnaires. The presence of the following diseases was defined as an answer of “yes” to questions regarding whether the participants had ever been diagnosed by a physician: (1) hypertension, (2) DM, (3) myocardial infarction, (4) angina, (5) stroke, (6) hypercholesterolemia, (7) dyslipidemia, (8) osteoarthritis, (9) rheumatoid arthritis, (10) pulmonary tuberculosis, (11) asthma, (12) thyroid-related disease, (13) major depressive disorder, (14) kidney failure, (15) hepatitis B, (16) hepatitis C, (17) liver cirrhosis, (18) gastric cancer, (19) hepatic cancer, (20) colorectal cancer, (21) breast cancer, (22) cervical cancer, and (23) lung cancer. The total breastfeeding duration was defined based on a questionnaire in which female participants responded as the total duration of breastfeeding during their lifetime. The “average breastfeeding duration” was calculated using the following formula: average breastfeeding duration = (total breastfeeding duration) / (number of breastfed children).

**Table 1:** Baseline characteristics of the study population

Variables	DM group (*n* = 2,248)	Control group (*n* = 13,691)	*p*-value
Age at enrollment (years)	67.9 (± 8.2)	63.2 (± 9.0)	< 0.0001
Body mass index (kg/m^2^)	25.2 (± 3.5)	24.1 (± 3.2)	< 0.0001
Waist circumference (cm)	86.5 (± 9.3)	81.8 (± 90)	< 0.0001
Systolic blood pressure (mmHg)	129.3 (± 17.2)	124.8 (± 17.8)	< 0.0001
Diastolic blood pressure (mmHg)	72.3 (± 9.6)	75.8 (± 9.8)	< 0.0001
Breastfeeding			
Average breastfeeding duration (month)	15.5 (± 9.7)	12.81(± 9.2)	< 0.0001
Breastfeeding duration group			< 0.0001
No breastfeeding	161 (7.2%)	1,383 (10.1%)	
< 12 months	362 (16.1%)	3,676 (26.9%)	
≥ 12 months	1,725 (76.7%)	8,632 (63.1%)	
Obstetric characteristics			
Age at menarche (years)	15.5 (± 2.03)	15.3 (± 2.0)	< 0.0001
Age at menopause (years)	49.0 (± 5.5)	49.2 (± 4.9)	0.047
Married	2,248 (100.0%)	13,680 (99.9%)	0.603
Gravidity	5.1 (± 2.4)	4.5 (± 2.1)	< 0.0001
Age at first delivery (years)	23.3 (± 3.5)	24.2 (± 3.6)	< 0.0001
Age at last delivery (years)	29.8 (± 4.6)	29.6 (± 4.4)	0.014
Prevalence of medical diseases			
Hypertension	1,524 (67.8%)	4,894 (35.8%)	< 0.0001
Dyslipidemia	1,216 (54.1%)	3,634 (26.5%)	< 0.0001
Stroke	145 (6.5%)	347 (2.5%)	< 0.0001
Myocardial infarction	65 (2.9%)	110 (0.8%)	< 0.0001
Kidney failure	30 (1.3%)	74 (0.5%)	< 0.0001
Liver cirrhosis	15 (0.7%)	42 (0.3%)	0.008
Biochemical profile at enrollment			
Fasting glucose (mg/dL)	134.7 (± 40.7)	97.7 (± 14.4)	< 0.0001
HbA1c	7.3 (± 1.3)	5.8 (± 0.5)	< 0.0001
Total cholesterol (mg/dL)	175.2 (± 38.8)	202.2 (± 37.3)	< 0.0001
HDL cholesterol (mg/dL)	47.1 (± 11.2)	52.2 (± 12.1)	< 0.0001
Triglycerides (mg/dL)	148.3 (± 85.1)	128.6 (± 80.8)	< 0.0001
LDL cholesterol (mg/dL)	100.0 (± 33.7)	124.6 (± 33.7)	< 0.0001
Family history			
Paternal family history of diabetes mellitus	153 (8.5%)	565 (4.8%)	< 0.0001
Maternal family history of diabetes mellitus	340 (17.8%)	1,068 (8. 6%)	< 0.0001
Socioeconomic and lifestyle characteristics			
Education level			< 0.0001
Elementary school and below	1,469 (65.4%)	6,475 (47.3%)	
Middle school	335 (14.9%)	2,297 (16.8%)	
High school	334 (14.9%)	3,366 (24.6%)	
College and above	109 (4.9%)	1,540 (11.3%)	
Household income			< 0.0001
Low	961 (43.0%)	3,976 (29.2%)	
Medium-low	601 (26.9%)	3,565 (26.2%)	
Medium-high	384 (17.2%)	3004 (22.1%)	
High	287 (12.9%)	3,062 (22.5%)	
Economic activity	709(31.5%)	6,158(45.0%)	< 0.0001
Residential areas			0.267
Urban	1,684 (74.9%)	10,404 (76.0%)	
Rural	564 (25.1%)	3,287 (24.0%)	
Frequency of drinking per year			< 0.0001
Never	832 (37.3%)	3,649 (26.8%)	
Have not drunk in the last 1 year	564 (25.3%)	2,862 (21.0%)	
Less than once a month	436 (19.5%)	3,246 (23.8%)	
Once a month	141 (6.3%)	1,244 (9.1%)	
2–4 times a month	171 (7.7%)	1,711 (12.6%)	
2–3 times a week	54 (2.4%)	653 (4.8%)	
≥ 4 times a week	34 (1.5%)	269 (2.0%)	
Smoking status			0.020
Smoker	84 (3.8%)	446 (3.3%)	
Ex-smoker	92 (4.1%)	425 (3.1%)	
Non-smoker	2,056 (92.1%)	12,767 (93.6%)	
Weekly weight training routines			< 0.0001
0 day	2,019(89.8%)	11,485 (83.9%)	
1 day	28 (1.3%)	366 (2.7%)	
2 days	40 (1.8%)	478 (3.5%)	
3 days	43 (1.9%)	466 (3.4%)	
4 days	25 (1.1%)	238 (1.7%)	
≥5 days	93 (4.1%)	656 (4.8%)	
Stress awareness			< 0.0001
Feel a great deal of stress	126 (5.7%)	624 (4.6%)	
Feel much stress	439 (19.7%)	2,616 (19.2%)	
Feel some stress	1,085 (48.6%)	7,618 (55.9%)	
Feel almost no stress	582 (26.1%)	2,776 (20.4%)	
Feeling depression in last 1 year	265 (11.8%)	1,523 (11.1%)	0.340

*DM* Diabetes mellitus, *HbA1c *Hemoglobin A1c, *LDL *Low-density lipoprotein, *HDL *High-density lipoprotein

Biochemical profiles, including HbA1c, fasting glucose, high-density lipoprotein (HDL), total cholesterol, and triglyceride (TG), were measured at the time of the survey. The measured low-density lipoprotein (LDL)-C was used for this study for subjects whose LDL-C was measured directly, and when it was not measured and serum TG levels were < 400 mg/dL, LDL-C was calculated using the Friedewald formula.

The characteristics of women with DM were compared to those of women without. To evaluate the association between breastfeeding and DM, the study population was divided into three groups by average breastfeeding duration: no breastfeeding group, < 12 months breastfeeding group, and ≥ 12 months breastfeeding group. The risk of DM was compared among the three groups.

### Statistical analyses

The variables were presented as mean values (standard deviation) or numbers (percentages). The chi-square test or Fisher’s exact test was used to compare categorical variables, and the t-test was used to compare continuous variables. Multivariable logistic regression analysis was performed to evaluate the risk of DM according to breastfeeding duration. The results from logistic regression are presented as odds ratios (OR), adjusted odds ratio (aOR), and 95% confidence intervals (95% CI). The < 12 months breastfeeding group was used as the reference group. For multivariate logistic regression analysis, covariates included sociodemographic characteristics (age at enrollment, body mass index (BMI), household income, areas of residence, occupation, and education level), obstetric characteristics (gravidity, age at first delivery, age at last delivery, age at menarche, and age at menopause), medical diseases (hypertension, myocardial infarction, angina, stroke, hypercholesterolemia, hyperlipidemia, osteoarthritis, rheumatoid arthritis, pulmonary tuberculosis, asthma, thyroid-related disease, major depressive disorder, kidney failure, hepatitis B, hepatitis C, liver cirrhosis, gastric cancer, hepatic cancer, colorectal cancer, breast cancer, cervical cancer, and lung cancer), family history, and socioeconomic and lifestyle characteristics (history of smoking, alcohol consumption, weekly weight training routines, stress awareness, and feeling depression within a year).

To evaluate the association between the average breastfeeding duration and DM, a machine-learning method was used. An artificial neural network, decision tree, logistic regression, naïve Bayes, random forest, and support vector machine were used to predict DM. Data on 15,939 observations with full information were divided into training and validation sets in a 70:30 ratio (11,157:4,782). The area under the receiver-operating-characteristic curve and accuracy (the ratio of correct predictions among the 4,782 observations in the validation set) were employed as the standards for model validation. The random-forest variable importance, the contribution of a certain variable to random-forest performance (accuracy), was used to examine the major predictors of DM. The importance of the random-forest variable was used to investigate the main factors of HbA1c. Variable importance provides the explanatory ability of the contribution of a specific variable to the outcome variable. Therefore, variable importance indicated important variables that had a strong association with the outcome, which was DM in the current study [21]. Then, random-forest Shapley Additive Explanations (SHAP) values were derived to examine the direction of association between DM and its major predictor. The SHAP value of a particular predictor for a particular observation measures the difference between what the model (random forest) predicts the probability of DM for the observation with and without the predictor (https://github.com/shap/shap). The inclusion of a predictor (average breastfeeding duration) into machine learning (random forest) will decrease or increase the probability of the dependent variable (DM). The SHAP values are skewed for the max value hence it can be concluded that there is a positive association between the average breastfeeding duration and DM.

The SHAP approach has a specific advantage compared to linear or logistic regression: the former incorporates all realistic scenarios unlike the latter. Let us make a simplistic assumption that there are three predictors of DM, i.e., BMI at enrollment, age at enrollment and the average breastfeeding duration. As explained above, the SHAP value of the average breastfeeding duration for DM for a particular participant is the gap between what machine learning predicts for the probability of DM with and without the average breastfeeding duration for the participant. Precisely speaking, the SHAP value for the participant is the average of the following four possible situations for the participant: (1) BMI at enrollment excluded, age at enrollment excluded; (2) BMI at enrollment included, age at enrollment excluded; (3) BMI at enrollment excluded, age at enrollment included; and (4) BMI at enrollment included, age at enrollment included [22]. That is, the SHAP value incorporates the outcomes of all possible sub-group analyses, which are missing in linear or logistic regression with an unrealistic presumption of *ceteris paribus*, i.e., “all the other variables staying constant”. Random split and analysis were repeated 50 times and averaged for external validation. R-Studio 1.3.959 (R-Studio Inc., Boston, United States) and Python 3.52 (CreateSpace: Scotts Valley, United States) were employed for the analysis between September 1, 2022-Ocober 30, 2022.

### Ethics

This study was approved by the Institutional Review Board (IRB) of the Korea University Anam Hospital (K2023-1954-001). The IRB waived the requirement for informed consent due to the anonymization of participant information. This study followed the Strengthening the Reporting of Observational Studies in Epidemiology (STROBE) guidelines [23].

## Results

Among the 15,946 participants, 2,248 (14.1%) women had DM and 14,402 (90.3%) had a history of breastfeeding. The study population was divided into two groups: the diabetes group consisted of women with a history of DM and the control group consisted of those without. The baseline characteristics of the study groups are presented in Table 1. The DM group included fewer women with a history of breastfeeding < 12 months (DM group vs. control group; 16.1% vs. 26.9%), but more women with a history of breastfeeding ≥ 12 months (DM group vs. control group; 76.7% vs. 63.1%) and with no history of breastfeeding (DM group vs. control group; 7.2% vs. 10.1%) than the control group. Women in the DM group delivered more children than those in the control group (DM group vs. control group; 5.1 vs. 4.5). More comorbidities were observed in the women in the DM group than in the control group.

**Fig. 2:**
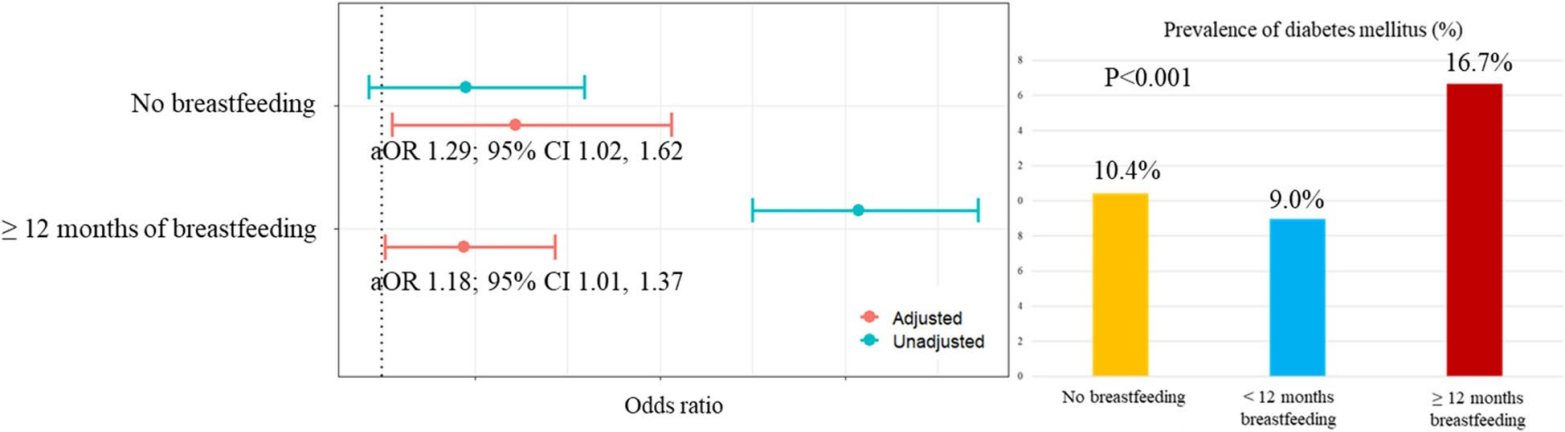
Risk of diabetes mellitus according to the average breastfeeding duration. Adjusted for sociodemographic characteristics (age at enrollment, BMI, household income, areas of residence, occupations, and education level), obstetric characteristics (gravidity, age at first delivery, age at the last delivery, age at menarche, and age at menopause), medical diseases (hypertension, myocardial infarction, angina, stroke, hypercholesterolemia, hyperlipidemia, osteoarthritis, rheumatoid arthritis, pulmonary tuberculosis, asthma, thyroid-related disease, major depressive disorder, kidney failure, hepatitis B, hepatitis C, liver cirrhosis, gastric cancer, hepatic cancer, colorectal cancer, breast cancer, cervical cancer, and lung cancer), family histories, and socioeconomic and lifestyle characteristics (history of smoking, alcohol consumption, weekly weight training routines, stress awareness, and feeling depression within one year)

The prevalence of DM was lowest in the < 12 breastfeeding group (no breastfeeding vs. < 12 months breastfeeding vs. ≥ 12 months breastfeeding: 161 (10.4%) vs. 362 (9.0%) vs. 1,725 (16.7%), *p* < 0.001) (Fig. 2). HbA1c and fasting glucose level measured at enrollment were also lowest in < 12 breastfeeding group (HbA1c: No breastfeeding vs. < 12 months breastfeeding vs. ≥ 12 months breastfeeding; 5.9% vs. 5.9% vs. 6.1%, *p* < 0.001, respectively; fasting glucose: No breastfeeding vs. < 12 months breastfeeding vs. ≥ 12 months breastfeeding; 101.1 mg/dL vs. 99.9 mg/dL vs. 104.4 mg/dL, respectively; *p* < 0.001). After adjustment for covariates, the risk of DM was increased in both, the no breastfeeding group (aOR 1.29; 95% CI 1.29, 1.62) and the ≥ 12 months of breastfeeding group (aOR 1.18; 95% CI 1.01, 1.37) compared to that in the < 12 months breastfeeding group. The protective effect of breastfeeding < 12 months on the risk of DM remained significant when analyzed the association using no breastfeeding group as a reference group (aOR 0.78; 95% CI 0.62, 0.98) (Supplementary Table 2) In addition, the association between the total breastfeeding duration and DM was evaluated. The risk of DM decreased in the < 12 months breastfeeding group compared to the no breastfeeding group, but a trend was observed where the protective effects diminished in the > 12 months breastfeeding group (aOR 2.61; 95% CI 1.00, 2.96), although the protective effect did not reach statistical significance in the < 12 months breastfeeding group (aOR 0.53; 95% CI 0.60, 1.07) (Supplementary Table 3).

The performance measures of the six machine-learning models for DM are presented in Table 2. The accuracy and area under the receiver-operating-characteristic curve (AUC) were used as performance measures. The performance measures of the random forest and logistic regression were the best across the board. Their respective accuracy results were 0.93 and 0.91 respectively, and their corresponding AUC results were 0.95 and 0.93. Among the 59 variables, we extracted the top 15 variables that had the greatest association with the prediction of DM using variable importance (Table 3).

**Table 2:** Model performance for diabetes mellitus: accuracy and AUC

Model	Accuracy	AUC
Logistic Regression	0.91	0.93
Decision Tree	0.89	0.78
Naïve Bayes	0.82	0.87
Random Forest	0.93	0.95
Support Vector Machine	0.90	0.91
Artificial Neural Network	0.88	0.66

*Abbreviation: AUC *Area under the receiver-operating characteristic curve

**Table 3:** Random forest feature importance (top 15 variables)

**(a) Random-forest feature importance of prediction model for diabetes mellitus**		
**Rank**	**Variables**	**Variable importance**
1	HbA1c	0.26
2	Fasting glucose	0.18
3	LDL	0.05
4	Total cholesterol	0.05
5	HDL	0.03
6	Waist circumference	0.03
7	DBP	0.03
8	Triglyceride	0.03
9	BMI at enrollment	0.03
10	SBP	0.02
11	Age at enrollment	0.02
12	Age at menopause	0.02
13	Age at last delivery	0.02
14	Age at first delivery	0.02
15	Total breastfeeding duration	0.02
**(b) Random-forest feature importance of prediction model for HbA1c**		
**Rank**	**Variables**	**Variable importance**
1	Diabetes mellitus	2757
2	BMI at enrollment	543
3	Age at enrollment	350
4	Age at menopause	307
5	Age at last delivery	275
6	Frequency of alcohol consumption	269
7	Age at menarche	255
8	Age at first delivery	236
9	Total breastfeeding duration	229
10	Occupation	217
11	Gravidity	202
12	Average breastfeeding duration	179
13	Household income	169
14	Stress awareness	164
15	Breastfeeding duration group	150

*DBP* Diastolic blood pressure, *HbA1c *Hemoglobin A1c, *LDL *Low-density lipoprotein, *HDL *High-density lipoprotein, *SBP *Systolic blood pressure, *BMI *Body mass index, *HbA1c *Hemoglobin A1c

**Fig. 3:**
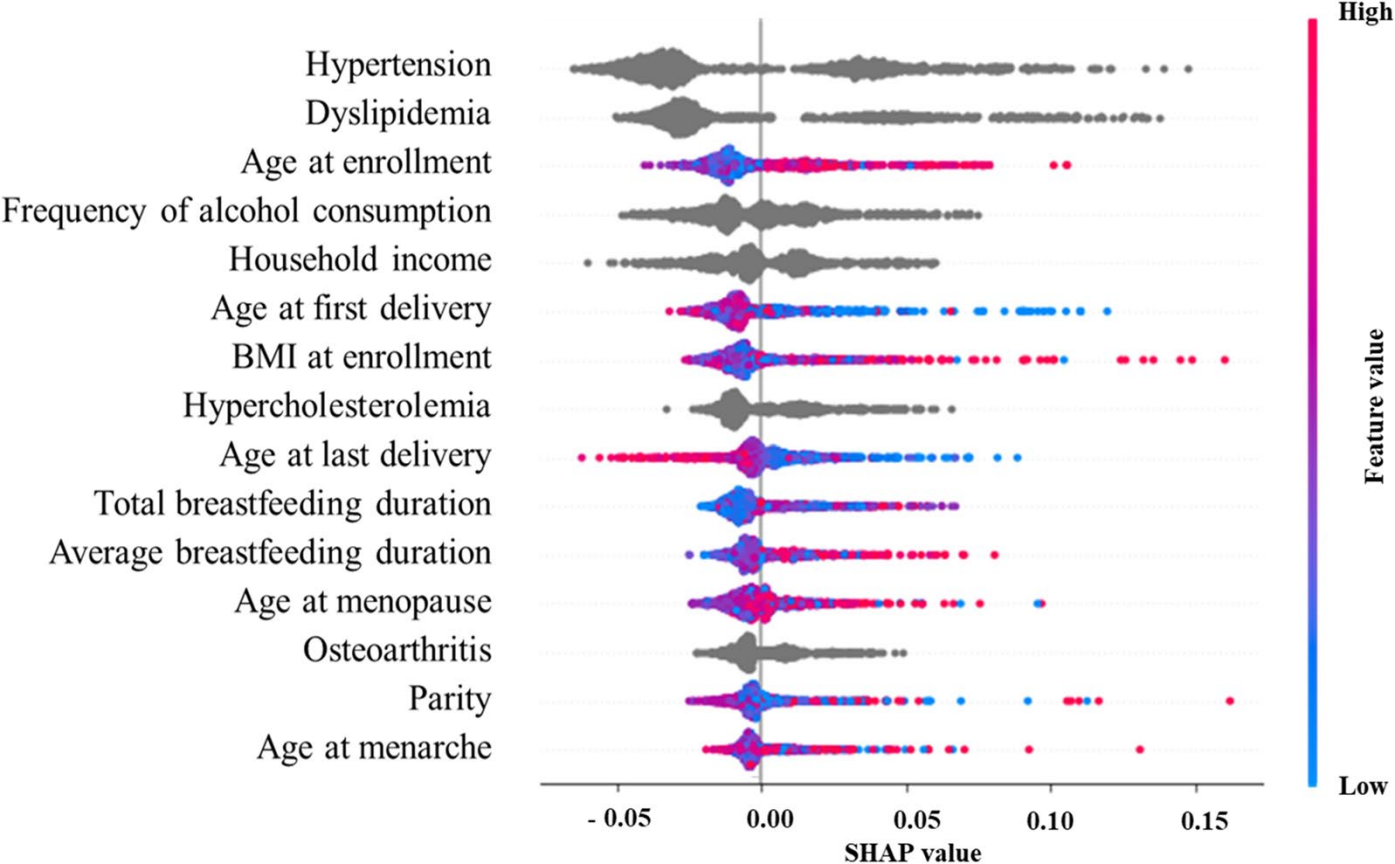
Random-forest SHAP summary plot

The top 15 predictors of DM were HbA1c, fasting glucose, LDL, total cholesterol, HDL, waist circumference, diastolic blood pressure, TG, BMI at enrollment, systolic blood pressure, age at enrollment, age at menopause, age at last delivery, age at first delivery, and total breastfeeding duration. The average breastfeeding duration was ranked the 17th. Here, the accuracy of the prediction model decreased by 1.5% if the values of the average breastfeeding duration were randomly permutated (or shuffled). The random-forest SHAP values are shown in Fig. 3. As explained above, the SHAP value of a particular predictor for a particular observation measures the gap between what the model (random forest) predicts the probability of DM for the observation with and without the predictor. The range of SHAP values for the average breastfeeding duration was from 0 to 0.05 (Supplementary Table 1). The SHAP values are skewed for the max value and there is a positive association between the average breastfeeding duration and DM (Fig. 3). In summary, the average breastfeeding duration was a significant predictor of DM, which has a strong association with DM, considering that the variable of average breastfeeding duration ranked in the upper half of the total variables in terms of both variable importance and SHAP value.

Furthermore, the prediction model for HbA1c was established by random forest, and the root mean square error over the mean was 0.11. The variables of both the average breastfeeding duration’ and breastfeeding duration groups were included in the top 15 variables of the prediction model (Table 3). Therefore, the average breastfeeding duration was associated with HbA1c level, similar to the prediction model of DM.

## Discussion

This large population-based study identified an optimal breastfeeding duration to prevent DM in women. The risk of DM in parous women was the lowest in women who had breastfed for less than an average of 12 months compared to that in parous women who had never breastfed or had breastfed for more than 12 months. The protective effect of breastfeeding on maternal DM increased during the first 12 months and weakened after 12 months of breastfeeding.

This study confirmed the protective effect of breastfeeding on DM. A possible mechanism for this protective effect is well established [8, 24–27]. Breastfeeding recuperates insulin resistance and glucose intolerance, which increase during pregnancy [8]. Breastfeeding has been associated with the proliferation of pancreatic β-cells in animal studies [24]. Likewise, the insulin concentration and glucose production rate were both lower in lactating rats than in non-lactating rats [25]. Moreover, milk production and breastfeeding consume additional energy and glucose, approximately 478 calories and 50 g of glucose per day [26, 27]. We found a weakened protective effect of breastfeeding for an average breastfeeding duration of more than 12 months. This finding is consistent with the previous studies showing that the risk of DM is reduced only during a short period of breastfeeding per child [9]. We suggest that prolongation of amenorrhea due to breastfeeding is a possible explanation for this finding. Previous research indicated the cessation of ovulation and menstruation during breastfeeding because of elevated level of prolactin and suppressed pulsatile [28–30]. Excessive breastfeeding duration beyond the period sufficient to restore the metabolic derangement caused by pregnancy is assumed to have a similar effect to prolonging the period of menopause, such as early menopause. Studies have demonstrated an association between early menopause and DM. Women who experience early or premature menopause have an increased risk of DM [31–33]. Likewise, premature ovarian insufficiency is also associated with an increased risk of DM [33]. Increased insulin resistance and pancreatic β-cell apoptosis and decreased insulin signaling through estrogen receptor α (ER α) caused by decreased estrogen levels contribute to an increased risk of DM [34].

This large population-based study found that the heightened beneficial effect of breastfeeding on DM in breastfeeding duration under 12 months. Likewise, machine learning analysis indicated a positive association between the average breastfeeding duration shown in and DM implies that an increased duration of breastfeeding does not decrease the risk of DM. Many studies have reported the beneficial effect of breastfeeding in preventing DM; however, several studies have focused on breastfeeding duration of less than 12 months [35–38]. Although some studies have analyzed the protective effect of breastfeeding for more than 12 months compared to no breastfeeding, the change in the breastfeeding effect on DM according to breastfeeding duration has not been analyzed [10, 39]. Moreover, through detailed interviews and questionnaires obtained by trained researchers, we adjusted for various risk factors for diabetes, including educational level, household income, smoking status, drinking and exercise habits, family history, and comorbidities. In addition, we comprehensively analyzed the association between breastfeeding duration and diabetes using machine learning, considering these various confounding factors. Machine learning is optimized for analyzing associations and establishing prediction models for diseases by considering various variables. To our knowledge, the current study is the first to analyze the breastfeeding effect on DM by machine learning. Furthermore, we analyzed the association between breastfeeding and DM using laboratory data (HbA1c), while most previous studies analyzed the breastfeeding effect on diabetes based on self-reported questionnaires [15, 19, 32, 35–38].

The strong association between breastfeeding and HbA1c found using machine-learning analysis reinforced the association between breastfeeding and DM. The limitation of this study was that the breastfeeding duration was based on questionnaires, and we did not consider gestational diabetes, which is a risk factor for diabetes in parous women. In addition, we could not assess the nutritional factors or eating habit as the confounding factors. Although KNHANES provides the database of food intake frequency surveys, we considered it challenging to accurately convert this database into variables for carbohydrate, fat, and protein calories. Furthermore, we were unable to distinguish between type I and Type II DM. However, considering the low prevalence of type I DM in Korea ranging from 0.017 to 0.021%, this limitation is unlikely to significantly impact the overall results [40].

In conclusion, breastfeeding is associated with a lower risk of DM. This protective effect persists for 12 months, then weakens after one year of breastfeeding. Breastfeeding is recommended by obstetricians and pediatricians in various societies, but the rate of breastfeeding is disappointing [41–43]. In South Korea, breastfeeding is encouraged as part of the national health plan by the Ministry of Health and Welfare, but the rate of breastfeeding has decreased over the decades [44, 45]. In the United States, the rate of breastfed infants at 1 year of age was only 35.0% in 2008, and the rate was only 33.0% in 2016–2018 [42, 44]. The results of this study validated the association between breastfeeding and the risk of DM development in women while also reinforcing the positive effects of breastfeeding on maternal health. Further research is needed to determine the optimal duration of breastfeeding in terms of lowering the risk of hypertension or other metabolic diseases.

### Supplementary Information

Supplementary Material 1.

## Data Availability

The data presented in this study are not publicly available. However, the data are available from the corresponding author upon reasonable request and under the permission of Korea National Health Insurance Service.

## Abbreviations

aOR: Adjusted odds ratio
CI: Confidence interval
DM: Diabetes mellitus
KNHANES: Korean National Health and Nutrition Examination Surveys
OR: Odds ratio
SHAP: Shapley additive explanations
