# Supplementary material for: Association between breastfeeding duration and diabetes mellitus in menopausal women: a machine-learning analysis using population-based retrospective study
Source: Int Breastfeed J. 2024 May 14;19:33. doi: 10.1186/s13006-024-00642-z (PMC11092012; doi:10.1186/s13006-024-00642-z)
Supplement: Supplementary file 1 — Supplementary Material 1. [file 13006_2024_642_MOESM1_ESM.docx]

**Supplementary Table 1. Random-forest SHAP value (top 15 variables)**

| **Rank** | **Variables** | **Minimum** | **Maximum** | **Mean** |
| --- | --- | --- | --- | --- |
| 1 | Hypertension | -0.065 | 0.148 | -0.0013 |
| 2 | Dyslipidemia | -0.050 | 0.138 | -0.0012 |
| 3 | Age at enrollment | -0.023 | 0.049 | -0.0003 |
| 4 | Frequency of alcohol consumption | -0.002 | 0.091 | -0.0003 |
| 5 | Household income | -0.002 | 0.131 | -0.0002 |
| 6 | Age at first delivery | -0.048 | 0.075 | -0.0002 |
| 7 | BMI at enrollment | -0.032 | 0.120 | -0.0002 |
| 8 | Hypercholesterolemia | -0.016 | 0.043 | -0.0002 |
| 9 | Age at last delivery | -0.005 | 0.106 | -0.0002 |
| 10 | Total breastfeeding duration | -0.002 | 0.073 | -0.0002 |
| 11 | Average breastfeeding duration | -0.003 | 0.054 | -0.0002 |
| 12 | Age at menopause | -0.061 | 0.060 | -0.0001 |
| 13 | Osteoarthritis | -0.004 | 0.076 | -0.0001 |
| 14 | Parity | -0.002 | 0.047 | -0.0001 |
| 15 | Age at menarche | -0.005 | 0.056 | -0.0001 |

BMI, body mass index

| **Supplementary Table 2.** **Logistic regression analysis of average breastfeeding duration and diabetes mellitus**   \|  \| Unadjusted OR \| \| \|  \| Adjusted OR \| \| \| \| --- \| --- \| --- \| --- \| --- \| --- \| --- \| --- \| \| OR \| 95% CI \| p-value \|  \| OR \| 95% CI \| p-value \| \| Average breastfeeding duration \|  \|  \| <.001 \|  \|  \|  \| 0.046 \| \| No breastfeeding \| Ref \|  \|  \|  \| Ref \|  \|  \| \| < 12 months \| 0.85 \| (0.70, 1.03) \|  \|  \| 0.78 \| (0.62, 0.98) \|  \| \| ≥ 12 months \| 1.72 \| (1.45, 2.04) \|  \|  \| 0.91 \| (0.73, 1.14) \|  \|   *Adjusted for sociodemographic characteristics (age at enrollment, sex, BMI, household income, areas of residence, occupations, and education level), health-related variables (family histories, history of smoking, alcohol consumption, weekly weight training routines, stress awareness, and feeling depression within a year), general obstetric characteristics (number of gravidity, age at primiparity, age at the last childbirth , age at menarche, and age at menopause), and presence of the diseases(hypertension, myocardial infarction, angina, stroke, hypercholesterolemia, hyperlipidemia, osteoarthritis, rheumatoid arthritis, pulmonary tuberculosis, asthma, thyroid-related disease, major depressive disorder, kidney failure, hepatitis B, hepatitis C, liver cirrhosis, gastric cancer, hepatic cancer, colorectal cancer, breast cancer, cervical cancer, and lung cancer) |
| --- | --- | --- | --- | --- | --- | --- | --- | --- | --- | --- | --- | --- | --- | --- | --- | --- | --- | --- | --- | --- | --- | --- | --- | --- | --- | --- | --- | --- | --- | --- | --- | --- | --- | --- | --- | --- | --- | --- | --- | --- | --- | --- | --- | --- | --- | --- | --- |

BMI, body mass index; OR, odds ratio

**Supplementary Table 3.** **Logistic regression analysis of total breastfeeding duration and diabetes mellitus**

|  | Unadjusted OR | | |  | Adjusted OR | | |
| --- | --- | --- | --- | --- | --- | --- | --- |
|  | OR | 95% CI | p-value |  | OR | 95% CI | p-value |
| Total breastfeeding duration |  |  | <.001 |  |  |  | <.001 |
| No breastfeeding | Ref |  |  |  | Ref |  |  |
| < 12 months | 0.86 | (0.68, 1.09) |  |  | 0.53 | (0.60, 1.07) |  |
| ≥ 12 months | 1.54 | (1.30, 1.82) |  |  | 2.61 | (1.00, 2.96) |  |

*Adjusted for sociodemographic characteristics (age at enrollment, sex, BMI, household income, areas of residence, occupations, and education level), health-related variables (family histories, history of smoking, alcohol consumption, weekly weight training routines, stress awareness, and feeling depression within a year), general obstetric characteristics (number of gravidity, age at primiparity, age at the last childbirth , age at menarche, and age at menopause), and presence of the diseases(hypertension, myocardial infarction, angina, stroke, hypercholesterolemia, hyperlipidemia, osteoarthritis, rheumatoid arthritis, pulmonary tuberculosis, asthma, thyroid-related disease, major depressive disorder, kidney failure, hepatitis B, hepatitis C, liver cirrhosis, gastric cancer, hepatic cancer, colorectal cancer, breast cancer, cervical cancer, and lung cancer).

BMI, body mass index; OR, odds ratio
